# Research on the mechanism how consumer expected regret affects market access for pirated information products

**DOI:** 10.1371/journal.pone.0343031

**Published:** 2026-02-20

**Authors:** Jia Xue, Peng Gao, Youshi He, Zhiwen Li, Hanyang Xu

**Affiliations:** 1 Management College, Jiangsu University of Technology, Changzhou, China; 2 Management College, Jiangsu University, Zhenjiang, China; 3 Nari Research Institute, Nari Group Corporation, Nanjing, China; FAME|GRAPE, POLAND

## Abstract

In the past, traditional supervision of pirated information products mostly adopted a post incident disposal model. Under the supervision of government departments and the continuous improvement of manufacturer product quality, the gap in quality and user experience between genuine and pirated products is expanding, thereby curbing the spread of pirated products. At present, with the emergence of cloud services, the spread of information products has changed from offline physical transactions to online digital services, and the sales model has changed from a one-time buyout to a subscription system of continuous payment. In this new context, there have been significant changes in the attributes of pirated information products and customer consumption patterns. The utility analysis model of quality preference and law enforcement supervision may not be well adapted in some cases. However, this study introduces regret theory and expected regret parameters to construct an impact analysis model on the market access of pirated information products, which is closer to the behavioral decision-making patterns of consumers in cloud service scenarios. The conclusion of this study indicates that customers’ sensitivity to expected regret directly affects the quality and price decisions of product manufacturers. The expected regret negative effect of pirated products also provides a more scientific basis for piracy regulation. Appropriate expected regret is not only beneficial for increasing legitimate consumer surplus and product manufacturer profits, but also promotes an increase in overall social welfare.

## 1. Introduction

In the era of digital economy, the forms and consumption patterns of information products are undergoing profound changes. Information products have shifted from closed and static physical products (such as e-books and CDs) to dynamically changing digital services (such as streaming media and software libraries). Consumer decision-making patterns have shifted from one-time purchases to long-term subscriptions, significantly increasing the uncertainty of consumer purchasing behavior. By 2025, the number of streaming media users in China had surpassed 1.085 billion, with long-video subscription users accounting for 64.61%. Subscription services have become the core business model for information products [1].

At present, digital technology and Internet media have greatly facilitated the spread of pirated information products. The concealment and convenience of pirated streaming services continue to impact the legitimate market [2]. The Motion Picture Association of America (MPA) has released the “2023 Global Content Piracy Threat Report”, which states that pirated streaming websites will have over 140 billion visits in 2022, with pirated film and television websites accounting for the highest proportion [3]. According to the “2024 Annual Development Report of China’s Drama Industry”, pirated videos have caused economic losses of over 20 billion yuan to legitimate platforms in recent years [4]. These cases not only reveal the huge scale of the piracy market, but also reflect that under technological empowerment, piracy has formed a sustainable alternative supply system. Even worse, pirated information products are continuously eroding the user base and profit margins of the legitimate market [5].

The emergence of subscription based services marks a shift in consumer purchasing behavior from one-time to persistent decision-making [6]. Subscription services originated from Software as a Service (SaaS) [7]. Subscription users do not need to pay high fees at once, and can access massive content at a lower cost, which significantly reduces the decision-making threshold for users [8]. For information product suppliers, subscription based systems can enhance product and service flexibility, reduce costs, and improve user engagement. It can reduce consumers’ tendency to choose piracy when making decisions through a carefully designed service ecosystem.

In the past, the analysis model for controlling pirated information products mainly focused on two dimensions: product quality and market supervision [5]. However, in the subscription model, consumers’ continued use decisions are often not solely based on objective product quality or rational calculations of enforcement costs, but are to a considerable extent influenced by emotional psychological factors. For example, when considering whether to switch to piracy channels, users may have strong expected regrets about the convenience, stability, or social recognition lost after giving up legitimate services.

Expected regret is a forward-looking emotional response [7]. It refers to individuals making decisions based on their evaluation of different possible outcomes, anticipating the regret emotions that may arise in the future if a specific choice is made [7]. In the context of consuming pirated information products, expected regret mainly stems from the uncertainty assessment of the quality and value of information products. This emotion includes two aspects: one is the concern about the quality risks, moral pressure, or lack of social identity that may be faced after choosing piracy (i.e., “action regret”); The second is the anticipated disappointment (i.e., “psychological regret”) of missing out on exclusive content, high-quality services, or complete experiences provided by genuine products.

Some studies suggest that expected regret plays an important moderating role in consumer decision-making [6,7]. For example, taking film and television platform products as an example, consumers not only focus on the quality of the program itself, but also whether they can enjoy the priority viewing rights of the video will affect their purchasing decisions. In addition, taking music software as an example, consumers not only pay attention to the music content itself, but also have a significant impact on their purchasing decisions through personalized recommendations and emotional experience value. Both cases indicate that increasing users’ perception of potential losses after abandoning genuine products can amplify their expected regret of choosing piracy and enhance their willingness to purchase genuine products. Therefore, this study creatively incorporates the expected regret variable into the consumer decision model to more systematically explain users’ choice behavior between genuine and pirated products.

Another part of the research suggests that expected regret can also significantly affect product manufacturer decisions [9,10]. At present, the focus of cracking down on piracy by legitimate manufacturers has shifted from external regulation and content competition to refined prediction of user psychology. In the early era of cable television, addressing piracy mainly relied on improving program quality and strengthening signal supervision, but the cost was high and the effect was limited [5]. In the era of streaming media, legitimate platforms can predict the psychological nodes that users may regret based on their behavior data, and accordingly optimize service design. For example, legitimate streaming video platforms, based on user viewing habits, push reminders before key episodes are updated, reinforcing their premonition of “missing out on excitement if not renewed”. In addition, music software uses emotional marketing such as “annual music listening reports” to evoke users’ emotional attachment to music memories and increase their psychological cost of leaving the platform. Therefore, this study switched its research perspective from post regulation to pre prediction, analyzing the incentive effect of expected regret on product manufacturers’ decision-making.

Although the impact of expected regret on consumer and product manufacturer decisions is clear, the mechanism by which expected regret affects market access for pirated information products is complex. On the one hand, when consumers anticipate that using piracy may result in experience loss or moral pressure, expected regret may inhibit piracy behavior [7]; On the other hand, if the subscription price is too high or the content does not meet expectations, consumers may also foresee the “regret of spending money” after purchasing the genuine version, which may actually promote piracy [9,10]. This bidirectional effect makes expected regret a key variable that requires dynamic quantitative analysis. The expected regret measurement framework proposed by Jiang et al. (2016) provides a quantitative approach from three dimensions: probability of occurrence, utility loss, and individual sensitivity, laying a theoretical foundation for this study [6].

This study aims to explore in depth how expected regret systematically affects market access for pirated information products. The core of this research is to incorporate quantitative expected regret mechanisms (occurrence probability, utility loss, individual sensitivity) into the quality decision game model of information product manufacturers, and analyze how consumers’ expected regret suppresses the market entry of pirated products and the resulting changes in manufacturers’ quality strategies. This study attempts to answer the following core questions:

(1) How to quantify consumers’ expected regret utility in choosing between genuine and pirated products?
(2) Under what conditions does expected regret inhibit or promote piracy behavior?
(3) How does expected regret affect the optimal pricing, quality investment, and profit of legitimate platforms?
(4) Will the introduction of expected regret improve consumer surplus and social welfare?

Solving these problems has good practical significance and provides a new path for legitimate manufacturers to suppress piracy. Atanu and Debarbata’s research (2013) showed that the increase in piracy rates can be suppressed by improving the quality of genuine products and strengthening piracy supervision [5]. Existing research has not conducted comparative analysis on customer types [5,9]. However, the core of our research lies in analyzing the impact of individual sensitivity indicators on market access for pirated products. On the one hand, for price sensitive customers, excessive investment in product research and development by legitimate manufacturers to improve product quality will not significantly curb piracy. But product manufacturers can offer subscription services with low prices, short cycles, and high frequency. For example, iQiyi Film and QQ Music members have launched subscription services with different periods. The lower the subscription price, the higher the subscription frequency, and the longer the subscription period, the more significant the customer’s expected regret. This is consistent with our conclusion that the stronger the sensitivity to expected regret, the more significant the negative utility of expected regret. On the other hand, for quality sensitive users, legitimate manufacturers need to highlight the different quality of membership level services. The better the product quality, the higher the membership level. For example, the office software WPS has a membership system with different levels, and super members can enjoy more intelligent artificial intelligence services than ordinary members. If the membership level increases and the payment price increases, the expected regret utility will increase. This is also consistent with our conclusion that an increase in expected regret net utility will lead to an increase in the negative utility of choosing piracy, thereby suppressing the market entry of piracy. Therefore, for different types of customers, legitimate manufacturers can adopt different expected regret mechanisms to suppress piracy, which highlights the theoretical innovation of our research.

Also, this study provides new ideas for the regulation of pirated information product, which can shift from post-supervision to pre-monitoring. Market regulatory authorities can adopt distinct supervision based on different types of pirated products. For information products with high sensitivity to expected regret, regulatory authorities can increase regulatory investment appropriately to curb the market entry of piracy. For example, when it comes to the latest popular movies, audiences are highly sensitive to the expected regret of such movies. At this time, regulatory authorities can strengthen their crackdown on pirated films, which can significantly promote the increase of legal consumer surplus. However, for films with lower popularity, the audience’s sensitivity to the film’s expectations is relatively weak. In this case, simply strengthening law enforcement supervision will not promote the increase of legal consumer surplus. Therefore, the regulatory effectiveness has different impacts on suppressing market access for different types of pirated information products.

Considering the overall social welfare, there are also significant differences in our research. Current studies have shown that excessive enforcement efforts can lead to manufacturers lowering product quality, which can harm legitimate consumer surplus and ultimately result in a decline in overall social welfare [5]. However, our research conclusion differs from previous studies. The introduction of the expected regret mechanism not only indirectly increases the psychological cost of users choosing piracy, but also reduces the dependence of legitimate manufacturers on high enforcement fees. At the same time, quantitative evaluation of expected regret can help legitimate manufacturers attract more consumers, promote the increase of legitimate consumer surplus, and thus stimulate the rise of total social surplus. These findings not only provide a scientific basis for the application of expected regret mechanisms, but also propose new methods for regulating pirated information product.

## 2. Literature review

Although digital information technology has created innovation in information products, the problem of piracy has not been effectively solved. Scholars are continuously studying market access for pirated information products from different perspectives [5,10]. The common conclusion of these studies indicates that market access for pirated information products is related to the decisions of consumers, product manufacturers, and government regulatory agencies. Sundarajan (2004) found that a monopoly provider’s optimal pricing strategy can lead to a unified attitude of consumers’ higher product quality [11]. Besides, the decisions of consumers and government regulatory agencies directly affect the decisions of product manufacturers, including investment in product quality and pricing [6]. Unlike previous static research models, this study quantitatively analyzes how consumers will affect product manufacturers’ quality and price decisions, as well as market regulatory status, under different expected regret states. Therefore, the dynamic model in this study has more predictive power in advance.

Previous studies have shown that before 2015, consumers considered product quality preferences more when purchasing genuine or pirated information products [5]. The reason is that in the past, pirated information products mainly consisted of CDs, terminal software, games, or high-definition movies, which were considered one-time buyout products. Consumers are more concerned about the quality of the products they purchase, and do not have to worry about whether the value of the products will decrease in the future [5]. First, Atanu and Debarbata’s research demonstrated that consumer product quality preferences have a significant impact on market access for pirated information products. Second, Kim et al. (2018) suggested that technical means, such as software- and hardware-based protection, should be used to reduce piracy [12]. Third, Verkijika (2018) pointed out that in piracy and network externalities, different pricing strategies can be used to increase genuine profits [13]. Fourth, providing low-quality products, product bundling, and differentiated content delivery speed are important ways to combat piracy. Fifth, Dul et al. (2020) established a product protection strategy based on the piracy rate but not on the protection strategy [14]. They found that the cost of consumer piracy had an impact on the selection of the protection strategy [14]. Finally, Bergh et al. (2022) explored the impact of the free version strategy on piracy based on quality decisions [15]. To sum up, although previous studies have shown that consumer quality preferences do have a significant impact on market access for pirated information products, the overall social benefits have not been significantly improved. To address this issue, this study has shifted its research perspective from post analysis to pre prediction, thereby incorporating consumer expected regret into the important considerations for market access to pirated information products.

Another part of the research suggests that product manufacturers not only need to consider consumer demand, but also external pressures from market regulation [10]. There are significant differences between pirated information products and ordinary pirated products. Pirated information products are mainly sold online, which also increases the difficulty of government supervision [16]. In the Internet era, the network spread faster, leading to the spread of pirated information products faster. In regions where piracy is prevalent, government regulatory agencies need to increase their enforcement efforts on the piracy market, which may help promote the sales of genuine products, but may not necessarily lead to an overall increase in social benefits [17]. Under the high-intensity law enforcement of government departments, pirated and genuine products will fall into vicious price competition, and the surplus of legitimate consumers will decrease, accompanied by a decline in overall social benefits [18]. Therefore, this study believes that there is a lag in market regulation of pirated information products and the enforcement costs are too high. To solve the problem of external regulatory lag, it is necessary to construct a quantitative prediction model in advance. The endogenous expected regret utility indicator proposed in this study considers both economic value and timeliness.

According to existing research, current information products are vastly different from those of the past [19]. The relationship between product manufacturers and consumers is no longer a one-time transaction, but a continuous service relationship [20]. Consumer decisions are complex decisions of subscription and payment, and the service cycle of information products is longer, which is also more likely to be accompanied by consumer expected regrets. The dissemination speed and scope of new information products such as big data, cloud services, and streaming media are faster and wider, and consumers’ demand for such information products is also constantly increasing [14]. The subscription data of information products also provides scientific basis for product manufacturers’ quality and price decisions [21]. Product manufacturers can better understand consumers’ demand preferences from subscription data. Subscription services also create lasting transactional relationships between product manufacturers and consumers [22]. Research has shown that subscription services have become the mainstream service for information products and have also created better revenue for product manufacturers. From this, it can be seen that expected regret helps product manufacturers anticipate consumer purchasing psychology in advance, shifting from passive regulation to endogenous active control, resulting in a decrease in both enforcement costs and quality investment costs [23]. In other words, expected regret is an important tool for product manufacturers to improve efficiency. Overall, previous research has focused more on the negative effects of pirated information products. However, this study suggests that genuine product manufacturers should shift from passive competition to proactive action. The quantified expected regret probability model can not only save quality input costs for product manufacturers, but also increase legitimate consumer surplus and total social welfare.

## 3. Model description and assumptions

Considering a competitive information product market consisted of a genuine provider, pirated products providers and several consumers. The previous study by Atanu and Debarbata (2013) revealed that the decisions of consumers and product manufacturers have a significant impact on market access for pirated information products [5]. Although consumers’ quality preferences and law enforcement regulations have imposed constraints on pirated products, the overall social benefits have not been effectively improved. In the past, information product transactions were one-time buyouts, while now information products are mainly based on persistent subscription services, and consumer decisions are more uncertain [23]. The traditional quality price static model analysis can no longer accurately evaluate the dynamic psychological changes of consumers, so this study introduces a dynamic quantitative model of expected regret. Compared with the above previous research, this study has made significant comparisons in the following aspects: research background, key factors and economic benefits of consumer, supplier and market supervisor as shown in Table 1 for details [5].

**Table 1:** Difference between previous research (2013) and this research (2025).

	Consumer		Supplier		Market supervisor	
Year	2013	2025	2013	2025	2013	2025
Research background	one time buyout decision	continuous subscription decision	passive competition	proactive prediction	lower efficiency	higher efficiency
Key factor	product quality preference	expected regret	quality and price	expected regret, quality and price	supervision cost	social welfare
Subject decision-making	static analysis of product quality and price	dynamic analysis of consumer psychology	external intervention of consumers and market regulation	internal consumer psychological prediction	ex post supervision	pre supervision

(Note: Atanu and Debarbata’s research represents 2013)

After comparative analysis of the above research, this study found that consumer decisions and product attributes have undergone significant changes today. Considering the continuity and uncertainty of information product transactions, this study introduces dynamic expected regret probability, economic expected regret utility, and differentiated expected regret sensitivity indicators. This study constructed a dynamic quantitative model to compare and analyze the changes in quality and price decisions of product manufacturers and consumers with different expected regret states. After that, this research deeply consider under appropriate regulatory effectiveness whether it can effectively constrain market access for pirated information products and create an increase in overall social benefits.

**Fig 1:**
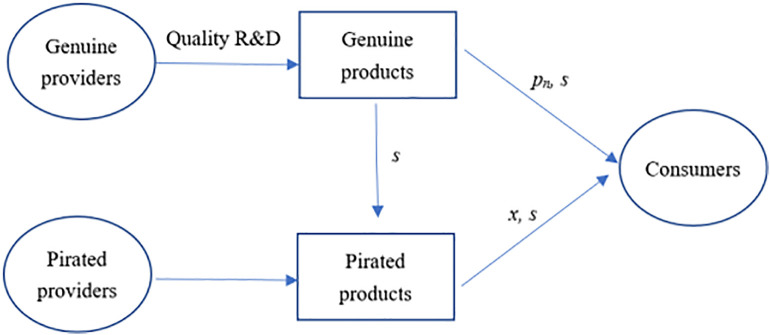
Market structure.

The genuine provider offers genuine products at a price *p* > 0 and incurs fixed R&D expenses on improving product quality *s*. Here, product quality can be evaluated in terms of the product’s stability and reliability as well as advancing and improving its function. However, piracy exists in the market and forms a competitive relationship with the genuine products. The price of the pirated product is *x,* which is directly determined by the piracy cost related to the enforcement level and viewed as an external variable, so *x* is also used to indicate the supervision level of piracy [8]. In addition, it is argued that the quality of pirated products is related to that of genuine products. However, there is a considerable gap in other features of the products such as their stability, reliability, and integrity, which consumers can effectively recognize. Therefore, consumers can quickly determine whether a product is genuine or pirated. A case in point is that according to the research released by ICC (International Chamber of Commerce), almost all users do not think that the reason they buy pirated products is that they cannot identify piracy, but they buy them because of price and other reasons [5,6]. The market structure is shown in Fig 1.

For better model presentation, all the symbols of the variables are listed in Table 2.

**Table 2:** Definitions of Explanatory Variables.

Variable	Definition
*p*	the price of genuine products
*x*	the price of pirated products, that is, the piracy supervision level [8]
*s*	the quality of genuine products
*c*	quality R&D cost coefficient of a genuine product
*D*_*g*_, *D*_*r*_	demand function of genuine/pirated products
μ	piracy rate, that is, the share of pirated products in the total information products
$\pi_{g}$,$\pi_{r}$	profit function of genuine/pirated products
*CS*	consumers’ surplus
*SW*	social welfare
*v*	consumers’ highest willingness to pay for quality
$\theta_{H}$,$\theta_{L}$	consumers’ high-/low-quality valuation of pirated products, where $0<\theta_{L}<\theta_{H}<1$
γ	the anticipated regret sensitivity coefficient, where 0<γ<1
θ	consumer’s expected quality valuation of piracy, where $\begin{array}{c} \theta =\left(\theta_{H}+\theta_{L}\right)/2 \end{array}$
*k*	the degree of difference in consumers’ valuation of the two types of piracy, where $k=\theta_{H}/\theta_{L}$
*U*_*g*_, *U*_*r*_	consumers’ utility when buying genuine/pirated products
$U_{f}$,$U_{c}$	utility of the forgone/chosen alternative

Here, consumer utility and anticipated regret are going to be described in detail. Similar to Kim et al. (2018) [12], it is assumed that consumers’ highest willingness for product quality (*v*) is uniformly distributed on (0,1). Each consumer knows his/her highest willingness for quality, but the manufacturer only knows the distribution. Hence, the net utility of the genuine product to a consumer is vs. Due to the lack of legal and technical support and piracy, there is a risk of a virus or system crash. Hence, although the products might be of the same quality, consumers’ valuation of the quality of pirated products is lower than that of genuine products [6]. θs is used to denote consumers’ valuation of the quality of pirated products, where θ (0<θ<1) refers to consumers’ valuation of piracy quality, representing consumer preference for piracy. Without losing generality, the heterogeneity of θ is considerd, that is, 50% of consumers are valued at $\theta_{H}$ and $\theta_{L}$ ($0<\theta_{L}<\theta_{H}<1$) [5,6].

Due to ethical and legal issues, it is assumed that it is not easy for the provider of pirated products to widely publicize the products, which leads to a vague valuation of the pirated products by consumers. Therefore, before product selection, consumers cannot confirm the actual quality valuation ($\theta_{H}$ or $\theta_{L}$) on their own. They can only make decisions based on the expected (average) valuation, i.e., $\left(\theta_{H}+\theta_{L}\right)/2$. As consumers use the product, their valuation of pirated products becomes precise. They may regret their previous decisions, which leads to anticipated regret before the next purchase. According to Jiang et al. (2017) [6], anticipated regret is reflected in the negative utility of the purchase process, and the following function can be used to measure it:


$$A.R.=-\gamma *prob\left(U_{f}>U_{c}\right)*\left(U_{f}-U_{c}\right),$$ (1)

where $U_{f}$ is the utility of the forgone alternative, and $U_{c}$ is the utility of the chosen alternative. In this paper, “forgone” refers to the transfer of forgone purchasing, that is, abandoning one product and choosing another. It does not include the case where both are abandoned. Similar assumptions were made in the study of Jiang et al. (2017) [6]. $prob\left(U_{f}>U_{c}\right)$ is the probability of anticipated regret. The formula shows that anticipated regret is used to measure the negative utility. $\gamma_{i}$ (i∈{f,c}) is the anticipated regret sensitivity coefficient, representing the degree of anticipated regret that consumers experience. The more sensitive consumers are to regret, the higher $\gamma_{i}$ is. Jiang et al. (2017) argued that consumers feel different levels of regret depending on whether they choose to forgo or purchase the product [6]. The above difference is ignored and setting $\gamma_{c}=\gamma_{f}=\gamma$, and 0<γ<1. Thus, it is known that the negative utility caused by anticipated regret consists of three parts—the probability of regret, the net utility loss when regret occurs, and the sensitivity of anticipated regret.

Other critical assumptions are as follows:

**Assumption 1.** Each consumer can only choose one product.

**Assumption 2.** The fixed R&D cost of a genuine product is a convex function of quality. Without losing generality, setting it as $\frac{\text{1}}{\text{2}}cs^{\text{2}}$, where *c* is the coefficient of the quality R&D cost of a genuine product. Moreover, other fixed costs are and assuming that the marginal cost of pirated products is zero. Many studies have adopted these assumptions [33].

**Assumption 3.** The information provider is a game leader, and consumers are followers. In the information provider’s decision-making, quality is at the strategic level, and price is at the tactical level. Thus, in terms of timeline, quality decisions are made before price decisions.

**Assumption 4.** Consumers have certain “ethical” attributes, implying that anticipated regret only happens before piracy purchasing and not before genuine purchasing.

## 4. Consumer decision-making process and demand function

Previous studies have found that consumers’ preferences for product quality and market regulation can have an impact on the decisions of genuine product manufacturers. The research findings of Atanu and Debarbata (2013) showed that when market regulation is weak, product manufacturers invest heavily in research and development costs to improve product quality and attract consumers, but this does not promote an increase in overall social benefits [5]. On this basis, this study also incorporates product quality and market regulatory enforcement costs into the model, but this study aims to address the issue of declining social benefits caused by pirated products. However, the special psychological behavior of consumer expected regret caused by the complexity of decision-making, regardless of the effectiveness of external regulation, whether the market is dominated by genuine or pirated products, will affect consumer net utility and thus affect consumer decision-making. In addition, external regulation and law enforcement require sustained investment in significant social costs. And expected regret is a defense mechanism that is naturally triggered by platform services and user psychology, with zero marginal cost. The platform only needs to continue investing in user experience, content library breadth, and community functionality to continuously strengthen this psychological “lock” and achieve the goal of “using products to combat piracy”. In addition, users who choose to stay on the platform due to expected regrets generate massive and authentic viewing behavior data, which becomes a valuable asset for the platform to predict demand and guide content investment (self-made or purchased). The expected regret mechanism also creates a positive cycle for genuine product manufacturers: more accurate content matching → higher user satisfaction and stickiness → stronger expected regret psychology → more stable user retention. In contrast, the piracy market is only a passive reaction to demand and cannot form such a data loop that drives innovation.

According to Assumption 1, each consumer can choose from one of the following three options: (i) buy the genuine product, (ii) obtain a pirated product, or (iii) forgo the use of the product completely. Consumers will compare the net utility before making a choice. With no anticipated regret, the utility of consumers buying genuine products is $U_{g}=sv-p$, and that of purchasing pirated products is $U_{r}=\frac{\theta_{H}+\theta_{L}}{\text{2}}sv-x$. Further consideration should be given to the negative utility of anticipated regret. According to formula (1), an actual situation in which anticipated regret occurs should be considered. When buying pirated products, type $\theta_{H}$ consumers obtain a more excellent net utility than type $\theta_{L}$ consumers. Thus, for consumers with an uncertain valuation, the probability of anticipated regret of buying pirated products, $prob\left(U_{f}>U_{c}\right)$, is 0.5. Thus, the utility of choosing (i.e., buying pirated products), $U_{c}$, is $\theta_{L}sv-x$, whereas the utility of forgoing (i.e., purchasing genuine products), $U_{f}$, is sv−p. In summary, the expected utility of consumers who have anticipated regret when buying pirated products is as follows:


$$\begin{array}{c} U_{r}=\frac{\theta_{H}+\theta_{L}}{\text{2}}sv-x-\gamma *prob\left(U_{f}>U_{c}\right)*\left(U_{f}-U_{c}\right)\\ \;=\frac{\theta_{H}+\theta_{L}}{\text{2}}sv-x-\frac{\gamma }{\text{2}}\left(\left(sv-p\right)-\left(\theta_{L}sv-x\right)\right)=\frac{\left(\theta_{H}\text{+}\theta_{L}-\gamma \left(1-\theta_{L}\right)\right)sv\text{+}\gamma p-\left(2+\gamma \right)x}{\text{2}} \end{array}$$ (2)

Using Assumption 4, the net utility of consumers purchasing genuine products is still $U_{g}=sv-p$, and the net utility of consumers who do not purchase is zero.

Then, consumers’ decision-making is discussed based on individual rationality (IR) and incentive compatibility (IC) constraints. Consumer buys genuine products if the following IR and IC conditions are satisfied:


$$U_{g}=sv-p\geq 0\to v\geq \frac{p}{s}\text{\textbackslash{}hspace\{0.17em}\;\;\;\;\;\;\;\;\}\;$$ (IR)

$$U_{g}>U_{r}\to sv-p\geq \frac{\left(\theta_{H}\text{+}\theta_{L}-\gamma \left(1-\theta_{L}\right)\right)sv\text{+}\gamma p-\left(2+\gamma \right)x}{\text{2}}\to v\geq \frac{\left(2+\gamma \right)\left(p-x\right)}{\left(2+\gamma -\theta_{H}-\left(1+\gamma \right)\theta_{L}\right)s}\text{\textbackslash{}hspace\{0.17em}\;\;\;\;\;\;\;\;\;\;\;\;\;\;\;\;\;\;\;\;\;\;\;\;\;\;\;\}$$ (IC)

Similarly, a consumer procures pirated products if the following IR and IC conditions are satisfied:


$$U_{r}=\frac{\left(\theta_{H}\text{+}\theta_{L}-\gamma \left(1-\theta_{L}\right)\right)sv\text{+}\gamma p-\left(2+\gamma \right)x}{\text{2}}\geq 0\to v\geq \frac{\left(2+\gamma \right)x-\gamma p}{\left(\theta_{H}\text{+}\left(1+\gamma \right)\theta_{L}-\gamma \right)s}\text{\textbackslash{}hspace\{0.17em}\;\;\;\;\;\;\;\;\;\;\;\;\}$$ (IR)

$$U_{r}>U_{g}\to sv-p\leq \frac{\left(\theta_{H}\text{+}\theta_{L}-\gamma \left(1-\theta_{L}\right)\right)sv\text{+}\gamma p-\left(2+\gamma \right)x}{\text{2}}\to v\leq \frac{\left(2+\gamma \right)\left(p-x\right)}{\left(2+\gamma -\theta_{H}-\left(1+\gamma \right)\theta_{L}\right)s}\text{\textbackslash{}hspace\{0.17em}\;\;\;\;\;\;\;\;\;\;\;\;\;\;\;\}$$ (IC)

**Fig 2:**
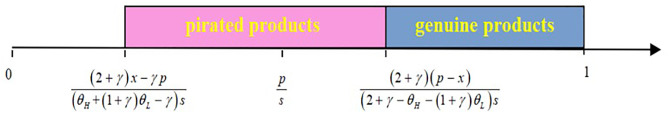
Schematic diagram of consumer choice in a competitive market.

**Fig 3:**
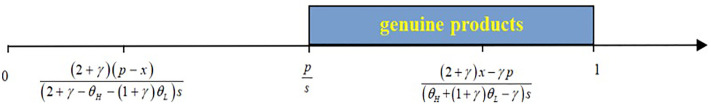
Schematic diagram of consumer decision-making under a monopoly market.

Comparing the three critical points, $v_{\text{1}}=\frac{p}{s}$, $v_{\text{2}}=\frac{\left(2+\gamma \mathrm{\backslash rightleft}(p-x\right)}{\left(2+\gamma -\theta_{H}-\left(1+\gamma \right)\theta_{L}\right)s}$, and $v_{\text{3}}=\frac{\left(2+\gamma \mathrm{\backslash rightleft}(p-x\right)}{\left(2+\gamma -\theta_{H}-\left(1+\gamma \right)\theta_{L}\right)s}$, the following two situations arise:

1. When $\;p>\frac{\left(2+\gamma \right)x}{\theta_{H}+\left(1+\gamma \right)\theta_{L}}$ $v_{\text{3}}<v_{\text{1}}<v_{\text{2}}$ is met, there are both pirated and genuine products on the market. Fig 2 shows consumers’ decisions.

1. When $\;p<\frac{\left(2+\gamma \right)x}{\theta_{H}+\left(1+\gamma \right)\theta_{L}}$ $v_{\text{2}}<v_{\text{1}}<v_{\text{3}}$ is met, there are no pirated products on the market, and consumers’ decisions are shown in Fig 3.

Based on the two situations, the market demand function of genuine products ($D_{g}\left(p,x,s\right)$) can be written as follows:


$$D_{g}\left(p,s\right)=\left\{ \begin{array}{c} @l@1-\frac{\left(2+\gamma \right)\left(p-x\right)}{\left(2+\gamma -\theta_{H}-\left(1+\gamma \right)\theta_{L}\right)s}\text{ if }p>\frac{\left(2+\gamma \right)x}{\theta_{H}+\left(1+\gamma \right)\theta_{L}}\mathrm{\backslash vspace}1.5mm\\ 1-\frac{p}{s}\;\;\;\;\;\;\;\;\;\;\;\;\;\;\;\;\;\;\;\;\;\;\;\;\;\;\;\;\;\;\;\;\;\;\;\;\;\;\;\;\;\;\;\;\;\;\;\;\;\;\;\;\;\;\;\;\;\;\;\;\;\;\;\;\;else \end{array}\right..$$ (3)

The demand for pirated products ($D_{r}\left(x,p,s\right)$) can be written as follows:


$$D_{r}\left(p,s\right)=\left\{ \begin{array}{c} @l@\frac{\left(2+\gamma \right)\left(p-x\right)}{\left(2+\gamma -\theta_{H}-\left(1+\gamma \right)\theta_{L}\right)s}-\frac{\left(2+\gamma \right)x-\gamma p}{\left(\theta_{H}\text{+}\left(1+\gamma \right)\theta_{L}-\gamma \right)s}\text{ if }p>\frac{\left(2+\gamma \right)x}{\theta_{H}+\left(1+\gamma \right)\theta_{L}}\mathrm{\backslash vspace}1.5mm\\ 0\;\;\;\;\;\;\;\;\;\;\;\;\;\;\;\;\;\;\;\;\;\;\;\;\;\;\;\;\;\;\;\;\;\;\;\;\;\;\;\;\;\;\;\;\;\;\;\;\;\;\;\;\;\;\;\;\;\;\;\;\;\;\;\;\;\;\;\;\;\;\;\;\;\;\;\;\;\;\;\;\;\;\;\;\;\;\;\;\;\;\;\;\;\;\;\;\;\;\;\;\;\;\;\;\;\;\;\;\;\;\;\;\;\;\;\;\;\;\;\;\;\;\;\;\;\;else \end{array}\right..$$ (4)

$\theta =\frac{\theta_{H}+\theta_{L}}{\text{2}}$ is used to denote consumers’ expected (average) valuation of the quality of a pirated product and use $k=\frac{\theta_{H}}{\theta_{L}}$ to denote the degree of difference in consumers’ valuation of the two types of piracy called consumer heterogeneity. It is obvious that k>1. Equations (3) and (4) can be written as follows:


$$D_{g}\left(p,s\right)=\left\{ \begin{array}{c} @l@1-\frac{\left(1+k\right)\left(2+\gamma \right)\left(p-x\right)}{\left(2\left(1-\theta \right)\left(1+k\right)+\left(1+k-2\theta \right)\gamma \right)s}\text{\textasciitilde{}}if\;p>\frac{\left(1+k\right)\left(2+\gamma \right)x}{2\theta \left(1+k+\gamma \right)}\mathrm{\backslash vspace}1.5mm\\ 1-\frac{p}{s}\text{\textbackslash{}hspace\{0.17em}\;\;\;\;\;\;\;\;\;\;\;\;\;\;\;\;\;\;\;\;\;\;\;\;\;\;\;\;\;\;\;\;\;\}\;\;\;\;\;\;\;\;\;\;\;\;\;\;\;\;\;\;\;\;\;\;\;\;\;\;\;\;\;\;\;\;\;\;\;\;\;\;\;\;\;\;\;\;\;\;\;\;\;\;\;\;else \end{array}\right.$$ (5)

$$D_{r}\left(p,s\right)=\left\{ \begin{array}{c} @l@\frac{\left(1+k\right)\left(2+\gamma \right)\left(p-x\right)}{\left(2\left(1-\theta \right)\left(1+k\right)+\left(1+k-2\theta \right)\gamma \right)s}-\frac{\left(1+k\right)\left(\left(2+\gamma \right)x-\gamma p\right)}{\left(2\theta \left(1+k\right)-\left(1+k-2\theta \right)\gamma \right)s}\text{\textasciitilde{}}if\;p>\frac{\left(1+k\right)\left(2+\gamma \right)x}{2\theta \left(1+k+\gamma \right)}\mathrm{\backslash vspace}1.5mm\\ 0\text{\textbackslash{}hspace\{0.17em}\;\;\;\;\;\;\;\;\;\;\;\;\;\;\;\;\;\;\;\;\;\;\;\;\;\;\;\;\;\;\;\;\;\;\;\;\;\;\;\;\;\;\;\;\;\;\;\;\;\;\;\;\;\;\;\;\;\;\;\;\;\;\;\;\;\;\;\;\;\;\;\;\;\;\;\;\;\;\;\;\;\;\;\;\;\;\;\;\;\;\;\;\;\;\;\;\;\;\;\;\;\;\;\;\}\;\;\;\;\;\;\;\;\;\;\;\;\;\;\;\;\;\;\;\;\;\;\;\;\;\;\;\;\;\;\;\;\;else \end{array}\right.$$ (6)

This shows that pirated products enter the market if the prices of genuine products are high. However, when the prices of genuine products are low, genuine products monopolize the market. The following settings: $A=\frac{\left(1+k\mathrm{\backslash rightleft}(2+\gamma \right)}{2\left(1-\theta \right)\left(1+k)+(1+k-2\theta \right)\gamma }$ and $B=\frac{\left(1+k\mathrm{\backslash rightleft}(2+\gamma \right)}{2\theta \left(1+k+\gamma \right)}$ are differentiated as follows:


$$\begin{array}{c} \frac{\partial A}{\partial \gamma }=\frac{-2\left(1+k\right)\left(k-1\right)\theta }{{\left(2\left(1-\theta \right)\left(1+k\right)+\left(1+k-2\theta \right)\gamma \right)}^{\text{2}}}<0\mathrm{\backslash vspace}1.mm\\ \frac{\partial B}{\partial \gamma }=\frac{\left(k-1\right)}{2\theta {\left(1+k+\gamma \right)}^{\text{2}}}>0 \end{array}$$ (7)

## 5. Price and quality decision analysis of information product providers

Based on the above analysis, the maximized profit of information product providers can be described as follows:


$$max\pi_{g}\;\left(p,s\right)=p\;\left(s\right)D_{g}\;\left(p,s\right)-\frac{\text{1}}{\text{2}}cs^{\text{2}}$$ (8)

According to the timeline described in Assumption 3, the backward induction method is used to solve the model. Theorem 1 is adopted to summarize the results of price decisions. For convenience, setting $f_{\text{0}}=\left(2B-1\right)A$ and $f_{\text{1}}=2B$.

**Theorem 1:** When consumers’ valuation of the quality of the pirated product ($\theta_{H},\text{\textbackslash{}hspace\{0.17em}\}\theta_{L}$), the sensitivity of anticipated regret (γ), and the price of the pirated product (*x*) are fixed, the formula for the optimal price of the genuine product is as follows:


$$p^{*}\;\;\left(s\right)=\left\{ \begin{array}{c} @l@\;\mathrm{\backslash vspace}1.5mm\\ \begin{array}{cc} @ll\frac{s}{2A}\text{+}\frac{x}{\text{2}} & \text{if }\frac{s}{x}\geq f_{\text{0}}\mathrm{\backslash vspace}1.5mm\\ Bx & \text{if }f_{\text{0}}>\frac{s}{x}\geq f_{\text{1}}\mathrm{\backslash vspace}1.5mm\\ \frac{s}{\text{2}} & \text{if }\frac{s}{x}<f_{\text{1}} \end{array}\text{\textbackslash{}hspace\{0.17em}\;\;\}\\ \;\;\;\;\;\;\;\;\; \end{array}\right.\;\;\;\;\;\;\;.$$ (9)

This indicates that in pure price decision-making, (1) when the quality–price ratio of the pirated product (i.e., $\frac{s}{x}$) is higher than a certain threshold ($f_{\text{0}}$), the optimal price of genuine products is monotonically increasingly insensitive to anticipated regret γ; (2) when the quality–price ratio is lower than the threshold $f_{\text{1}}$, γ does not affect the optimal price of the genuine product; (3) when the quality–price ratio is between two thresholds, an increase in γ increases the price of the genuine product.

Atanu and Debarbata (2013) emphasize that pirated products are competitors to genuine products, and quality and price are important means for product providers to counter pirated products [5]. Consumers are more focused on product quality preferences. However, due to the fact that subscription based information products are not one-time transactions, consumers cannot intuitively evaluate the future value of the product. Therefore, consumer expectations of regret will directly affect product pricing. And our research found (Theorem 1) that when the market is a non genuine complete monopoly, consumer expected regret will affect the pricing of information products.

In Theorem 1, the first condition represents a competitive market; the second is a monopoly market but is threatened by pirated products, and the third is a pure monopoly of genuine products. $\frac{s}{x}$ represents the ratio of information product quality and piracy supervision (in this study, it is referred to as the quality–cost ratio). As the quality–cost ratio of piracy increases, under a pure price decision, the market appears in three states in turn—piracy competition, piracy threat, and pure monopoly. As long as there is a threat of piracy, whether pirated products enter the market or not, anticipated regret will reduce consumers’ expected net utility of buying pirated products, increase the perceived utility difference between the two products, and help alleviate the competitive pressure on genuine products, which will lead to a high product price. However, when the quality of pirated products is low or the piracy price is high enough ($\frac{s}{x}<f_{\text{1}}$), genuine products will completely monopolize the market. Therefore, consumers do not need to make product choices, and the clear decision-making process will not produce anticipated regret.

Next, conducting the quality decision analysis. Based on the result of the price decision, the formula for the demand for genuine products (${D_{g}}^{*}\left(s\right)$) in terms of product quality is as follows:


$${D_{g}}^{*}\;\;\left(s\right)=\left\{ \begin{array}{c} @l@\frac{s+Ax}{2s}\text{ if }\frac{s}{x}>f_{\text{0}}\mathrm{\backslash vspace}1.5mm\\ 1-\frac{Bx}{s}\text{ if }f_{\text{0}}\geq \frac{s}{x}\geq f_{\text{1}}\mathrm{\backslash vspace}1.5mm\\ \frac{\text{1}}{\text{2}}\;\;\;\;\;\;\;\;\;\;\;\;\text{\textbackslash{}hspace\{0.17em}\;\}\;\;\;\;\;\text{if }\frac{s}{x}<f_{\text{1}} \end{array}\right.$$ (10)

Therefore, the profit objective function of information product providers can be expressed as $max\pi_{g}\left(s\right)=p^{*}\left(s\right){D_{g}}^{*}\left(s\right)-\frac{c}{\text{2}}s^{\text{2}}$, and it is a quadratic function of product quality *s*. And now the equilibrium result of the quality decision is obtained, which is summarized in Theorem 2.

**Theorem 2:** When setting $\rho_{\text{1}}\left(\gamma \right)=\frac{B{\left(\gamma \right)}^{\text{2}}}{c{\left(2B\left(\gamma \right)-1\right)}^{\text{3}}A{\left(\gamma \right)}^{\text{3}}},\rho_{\text{2}}\left(\gamma \right)=\frac{\text{1}}{8cB\left(\gamma \right)}$, the optimal quality level can be determined under the following conditions:

(1) When $x<\rho_{\text{1}}\left(\gamma \right)$, pirated products enter the market. Here, the information product provider’s optimal quality is $s^{*}=\tilde{s}$, where $\tilde{s}$ is the solution of the equation $G\left(s\right)=s^{\text{2}}-A{\left(\gamma \right)}^{\text{2}}x^{\text{2}}-4A\left(\gamma \right)cs^{\text{3}}=0$ and satisfies $\tilde{s}>f_{\text{0}}x$.
(2) When $\begin{array}{c} \rho_{\text{1}}\left(\gamma \right)\leq x\leq \rho_{\text{2}}\left(\gamma \right) \end{array}$, genuine products will monopolize the market, but when threatened by pirated products, the optimal quality is $s^{*}={\left(\frac{B^{\text{2}}x^{\text{2}}}{c}\right)}^{\frac{\text{1}}{\text{3}}}$.
(3) When $x>\rho_{\text{2}}\left(\gamma \right)$, genuine products will completely monopolize the market and the optimal quality $s^{*}=\frac{\text{1}}{4c}$.

This theorem reveals the effect of supervision on pirated products (i.e., the price of pirated products) under quality decisions. When the supervision (*x*) is less than the threshold determined by the sensitivity of consumers’ anticipated regret ($\rho_{\text{1}}\left(\gamma \right)$), pirated products will enter the market. However, the high cost of piracy will prevent it from entering the market when the supervision intensity is higher than$\rho_{\text{2}}\left(\gamma \right)$ (it can be easily found that $\rho_{\text{2}}\left(\gamma \right)>\rho_{\text{1}}\left(\gamma \right)$), where the genuine products will be in a monopoly and will not be threatened by pirated products. Here, consumers’ valuation of pirated products and sensitivity of anticipated regret will not affect information product providers’ quality decisions, so this situation will not be considered in the subsequent analysis. The impact of consumers’ anticipated regret on the entry of pirated products under the quality decision is studied, the relationship between $\rho_{\text{1}}\left(\gamma \right)$ andγ is explored, and the following Lemma is obtained:

**Lemma 1:** (1) When consumers’ piracy quality valuation is low (i.e., $\theta <3-\sqrt{\text{7}}$), an increase in γ reduces the price threshold of pirated products entering the market ($\rho_{\text{1}}\left(\gamma \right)$). (2) When consumers’ piracy quality valuation is high (i.e., $\theta >\frac{\left(9-3\sqrt{\text{7}}\right)}{\text{2}}\left(\frac{1+k}{2+k}\right)$), the price threshold of pirated products entering the market ($\rho_{\text{1}}\left(\gamma \right)$) increases with γ. (3) In other cases, with an increase in γ, the price threshold of pirated products entering the market ($\rho_{\text{1}}\left(\gamma \right)$) first increases and then decreases.

Atanu and Debarbata (2013) focused more on studying market access for pirated information products from the perspective of external regulation in the market where the product is located [5]. However, the lag in market regulation has resulted in a lack of overall social benefits, and product manufacturers have also been trapped in passive vicious price competition. Therefore, this study shifts from a post perspective to a pre perspective, where expected regret can help product manufacturers predict in advance and proactively evaluate the product market effectively.

**Fig 4:**
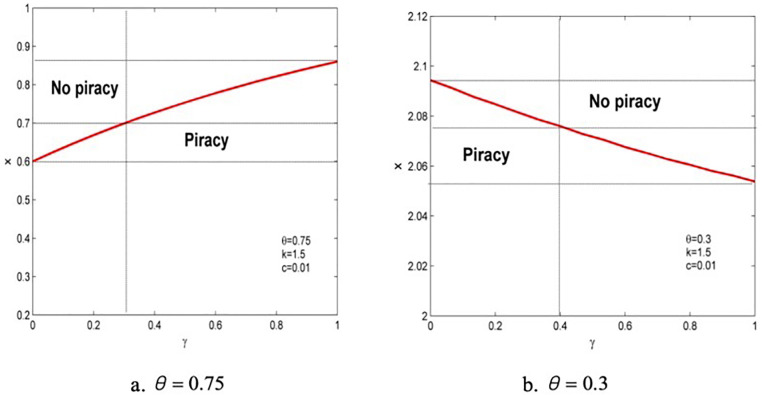
Area map of the effect of anticipated regret on piracy entry.

Lemma 1 shows the uncertainty of the effect of anticipated regret on the threshold of pirated products entering the market. Consumers’ quality valuation of piracy (θ) plays a role in regulating its entry. When θ is enormous, anticipated regret makes piracy supervision difficult, and vice versa. This is because, to a certain extent, anticipated regret is conducive to enhancing consumers’ perception of the difference in the quality of the two products when the valuation of pirated products is low, and quality difference perception will undoubtedly strengthen the market position of genuine products and reduce the pressure of external piracy supervision. However, as we can see from Fig 4, when consumers’ valuation of pirated products is high, the provider’s strategy of responding to anticipated regret will promote the entry of pirated products into the market, making it more challenging to regulate piracy.

Without losing generality, first, setting parameters c = 0.01 and k = 1.5 and then exploring the relationship between anticipated regret and the piracy supervision threshold. Based on the thresholds of low- and high-quality valuation obtained using Lemma 1, the two cases of θ=0.75 and θ=0.3 are used for the following analyses to arrive at a vivid conclusion, as shown in Fig 4. It is found that when $\begin{array}{c} x<min\left(\rho \left(0\right),\rho \left(1\right)\right) \end{array}$, pirated products will enter the market. When $\begin{array}{c} x>max\left(\rho \left(0\right),\rho \left(1\right)\right) \end{array}$, pirated products will not enter the market. However, when piracy supervision is moderate (i.e., $\begin{array}{c} min\left(\rho \left(0\right),\rho \left(1\right)\right)<x<max\left(\rho \left(0\right),\rho \left(1\right)\right) \end{array}$), anticipated regret will influence the entry strategy of the piracy market. When the quality valuation of piracy is low (θ=0.3), anticipated regret reduces the probability of piracy, and information providers can use anticipated regret as a competitive weapon against piracy threats to obtain profit. However, in the context of high-quality valuation of piracy (θ=0.75), anticipated regret can transform the market from a monopoly state to a pirate competition state, and it becomes the “breeding ground” for piracy. Therefore, Lemma 1 can be simplified as Lemma 2.

**Lemma 2:** (1) In the case of low piracy quality valuation, pirated products enter the market when γ is less than the threshold ${\rho_{\text{1}}}^{-1}\left(x\right)$. (2) In high piracy quality valuation, pirated products enter the market when γ is higher than the threshold. (3) Otherwise, the relationship between the piracy entry and γ remains uncertain.

Next, it sheds light on the change in the law about product quality. First, the impact of piracy supervision (*x*) on optimal quality s^*^ is examined and Lemma 3 is hence derived.

**Lemma 3:** When there is piracy in the market, $s^{*}$ is negatively correlated with *x*, and when there is no piracy in the market, $s^{*}$ is positively correlated with *x*.

Based on the research of Atanu and Debarbata (2013) [5], we further clarified the rational interval of legal regulation and compared and analyzed the decision changes of product manufacturers under different levels of expected regret sensitivity.

**Fig 5:**
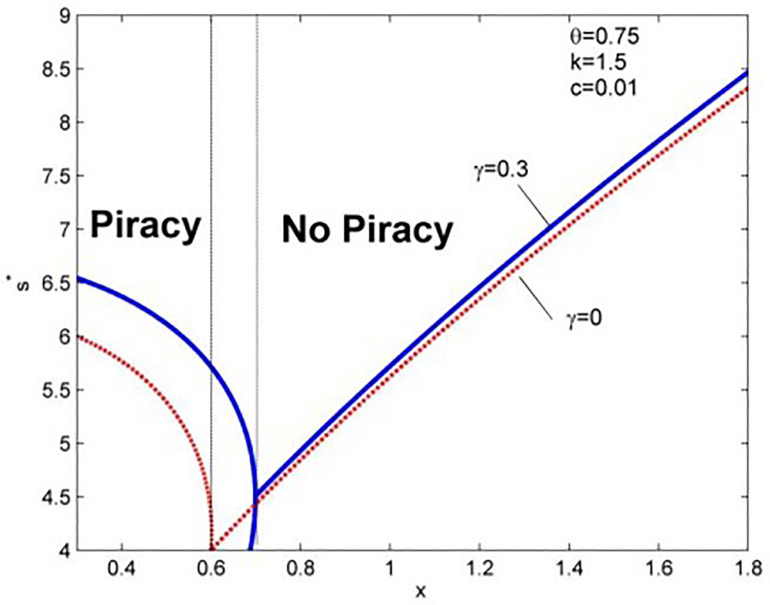
The impact of piracy supervision on optimal quality.

Lemma 3 and Fig 3 show that, unlike what most people expect, in the presence of piracy, strengthening piracy supervision reduces the quality of information products. When there is no piracy in the market, more substantial supervision can inspire providers to invest in quality R&D and obtain higher monopoly profits, which confirms the conclusion of Lahiri & Dey [24]. Fig 5 shows that the quality curve of γ=0.3 is always above that of γ=0, which makes us study the relationship between anticipated regret and the optimal quality level, and Lemma 4 is obtained.

**Lemma 4:** Whether there is piracy in the market or not, $s^{*}$ and $p^{*}$ are always positively related to γ.

**Fig 6:**
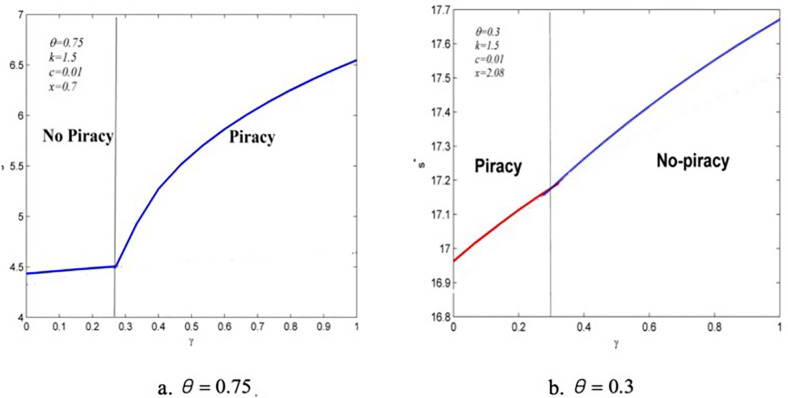
The relationship between optimal quality and anticipated regret.

Lemma 4 indicates that whether piracy enters the market or not, consumers’ anticipated regret increases the quality and price of information products. Fig 6 shows the result when θ\hspace{0.17em}=\hspace{0.17em}0.75 and θ\hspace{0.17em}=\hspace{0.17em}0.3 and piracy supervision is in the range of ($\begin{array}{c} min\left(\rho \left(0\right),\rho \left(1\right)\right) \end{array}$,$\begin{array}{c} max\left(\rho \left(0\right),\rho \left(1\right)\right) \end{array}$). To ensure the integrity of the conclusion, we divided it into low- (θ\hspace{0.17em=0.3}) and high-quality valuation (θ\hspace{0.17em=0.75}) situations and make sure that when γ changes between 0 and 1, there will be two situations—piracy enters the market or not. From Fig 6, when θ\hspace{0.17em=0.3}, the interval range of x is [2.05, 2.10], and when θ\hspace{0.17em=0.75}, the interval range of x is [0.6, 0.9]. If the value of x is not in the above range, we cannot derive complete conclusions. Thus, when θ\hspace{0.17em=0.75}, we use x = 0.7, and when θ\hspace{0.17em=0.3}, we use x = 2.08.

Interestingly, when θ=0.75, the optimal quality rises significantly faster when piracy exists than when there is no piracy. This proves that anticipated regret stimulates the entry of pirated products into the market (Lemma 2). Thus, the market changes from monopoly to competition. Here, the impact of quality differentiation is enormous, and anticipated regret will drive the provider to significantly increase quality R&D investment and supplement it with a price increase strategy to obtain stable profits. In case of piracy acceptance (θ=0.3), anticipated regret chases pirated products out of the market. Its impact on product quality is almost the same in the two markets, whether competition or monopoly. This is because the difference in consumers’ quality valuation of the two products is already considerable. Whether there is piracy in the market or not will no longer have a significant influence on the marginal effect of anticipated regret.

The previous study suggests that high-intensity enforcement may incentivize more consumers to choose genuine products, but does not significantly increase the revenue of product manufacturers [5]. And this is also the key issue we are studying, how to make the quantified expected regret mechanism promote the growth of social benefits. According to the case study data presented earlier, consumers purchasing subscription service products are more uncertain, but it significantly increases the revenue of product manufacturers. This is also consistent with our research conclusion that, under appropriate regulatory effectiveness, consumer expected regret is a catalyst that motivates product manufacturers to improve product quality and increase investment, regardless of whether piracy exists in the market.

Now, paying attention to an important question: how does the market piracy rate change with consumers’ anticipated regret? The piracy rate refers to the share of pirated products in the total number of products. It is an important indicator that reflects the status of piracy in a region. When it is a monopolistic market, the piracy rate is 0. Therefore, only the case of a competitive market is analyzed. Based on the demand function of Equations (3) and (4), the piracy rate can be expressed as follows:


$$\mu \left(\gamma \right)=1-\frac{D_{g}\left(\gamma \right)}{D_{g}\left(\gamma \right)+D_{r}\left(\gamma \right)}$$ (11)

Thus, Lemma 5 is obtained.

**Lemma 5:** The demand for genuine products $D_{g}\left(\gamma \right)$ is always negatively correlated with γ. The total demand $D_{g}\left(\gamma \right)+D_{r}\left(\gamma \right)$ is still strictly positively correlated with γ. The piracy rate μ(γ) is always strictly positively correlated with γ.

The study by Atanu and Debarbata (2013) emphasizes the differences in consumer preferences for quality, prompting product providers to differentiate between genuine and pirated product designs [5]. Because in 2013, most information products were bought out at once, consumers were more concerned about quality and did not need to consider whether the product value would change in the future. However, due to the rapid development of the Internet, the emergence of subscription services has extended the decision-making cycle of consumers, and consumers’ psychology will also change. Therefore, we also pay attention to the individual heterogeneity differences of consumers’ psychology, and the expected sensitivity index has well quantified the personality differences of consumers. If there is piracy in the market, the sensitivity to expected regret increases, the demand for pirated products increases, while the demand for genuine products decreases, leading to an increase in the market piracy rate. This also explains that although product providers’ decisions are influenced by quality and can promote differentiation in the quality of genuine products, it cannot avoid the emergence of pirated information products.

**Fig 7:**
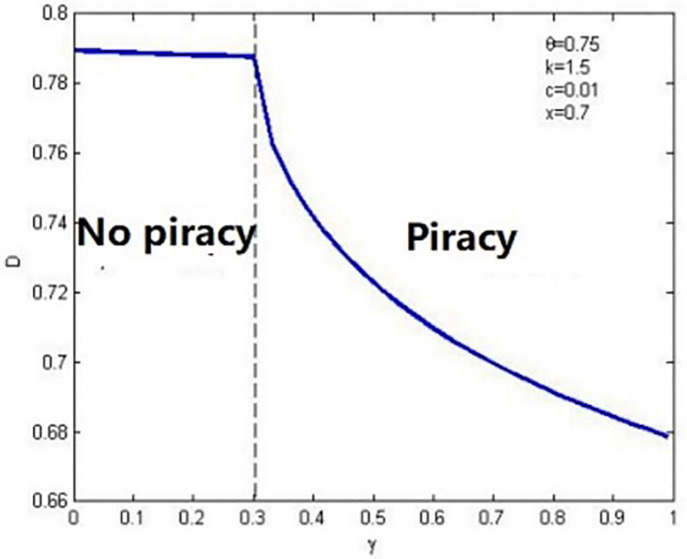
The relationship between the demand for genuine products and anticipated regret.

Lemma 5 clearly shows that the usual impression about the relationship between piracy rate and consumers’ anticipated regret does not hold. At a lower anticipated regret sensitivity, consumers’ demand for pirated products is high, and the demand for genuine products is low (Fig 7, which increases the piracy rate. This is due to the quality and price strategies of the provider. As stated in Lemma 4, consumers’ anticipated regret about purchasing pirated products stimulate providers to increase investment in quality R&D, thereby improving the quality of pirated products. So, consumers who did not purchase will enter the market to buy pirated products, whereas high prices may hinder those ready to buy genuine products. Thus, the provider’s quality strategy will strengthen the differentiated advantages of genuine products, but it is not conducive to curbing piracy. Therefore, clear product publicity is an effective way to reduce any ambiguity in consumers’ decision-making process, which can reduce consumers’ sensitivity to anticipated regret.

## 6. Consumer surplus, the profit of provider, and social welfare

This section investigates the consumer surplus, profit of provider, and social welfare by simulation due to the complexity of the results of the model. Lemma 2 shows that the effect of anticipated regret is regulated by consumers’ valuation of piracy quality θ, so we divided the scenarios into two—θ=0.75 and θ=0.3. Firstly, the “legal” consumer surplus of buying genuine products $CS_{L}$ and the total consumer surplus $CS_{T}$ are investigated. The former excludes users who buy pirated products. From the consumer utility diagrams presented in Figs 2 and 3, the expressions for legal consumer surplus can be obtained as follows:


$$CS_{L}=\left\{ \begin{array}{c} @l@\begin{array}{cc} @ll\int_{\frac{\left(1+k\right)\left(2+\gamma \right)\left(p-x\right)}{\left(2\left(1-\theta \right)\left(1+k\right)+\left(1+k-2\theta \right)\gamma \right)s}}^{\text{1}}\left(sv-p\right)dv\; & x<\rho_{\text{1}}\left(\gamma \right)\mathrm{\backslash vspace}1.5mm\\ \int_{\;\;\frac{p}{s}}^{\text{1}}\left(sv-p\right)dv\; & \;x>\rho_{\text{1}}\left(\gamma \right) \end{array}\;\\ \; \end{array}\right.$$ (12)

The expression for the total consumer surplus is as follows:


$$CS_{T}=\left\{ \begin{array}{cc} @ll@\int_{\frac{\left(1+k\right)\left(2+\gamma \right)\left(p-x\right)}{\left(2\left(1-\theta \right)\left(1+k\right)+\left(1+k-2\theta \right)\gamma \right)s}}^{\text{1}}\left(sv-p\right)dv\text{+}\;\int_{\frac{\left(1+k\right)\left(\left(2+\gamma \right)x-\gamma p\right)}{\left(2\theta \left(1+k\right)-\left(1+k-2\theta \right)\gamma \right)s}}^{\frac{\left(1+k\right)\left(2+\gamma \right)\left(p-x\right)}{\left(2\left(1-\theta \right)\left(1+k\right)+\left(1+k-2\theta \right)\gamma \right)s}}\left(\frac{\left(2\left(1+k\right)\theta -\left(1+k-2\theta \right)\gamma \right)sv\text{+}\left(\gamma p-\left(2+\gamma \right)x\right)\left(1+k\right)}{2\left(1+k\right)}\right)dv & \begin{array}{c} x<\rho_{\text{1}}\left(\gamma \right) \end{array}\\ \int_{\frac{p}{s}}^{\text{1}}\left(sv-p\right)dv\; & x>\rho_{\text{1}}\left(\gamma \right) \end{array}\right.$$ (13)

**Fig 8:**
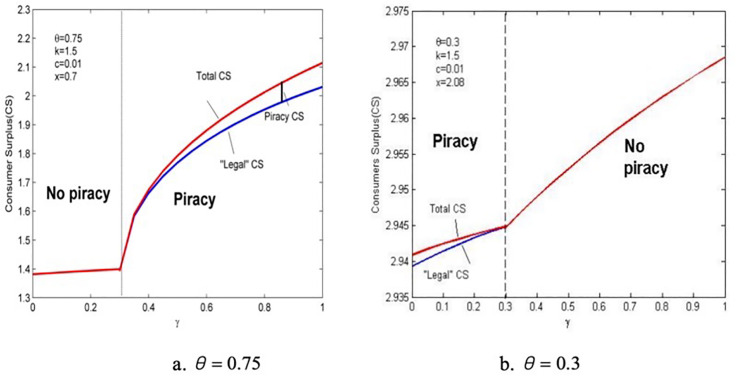
The impact of anticipated regret on consumer surplus.

Drawing the graphs of changes in the law about $CS_{L}$ and $CS_{T}$ using γ in the two scenarios, which are shown in Fig 8.

It is surprised to find that although anticipated regret is expected to be a negative utility, it does not reduce the consumer surplus under the influence of the provider’s price and quality strategies, whether illegal users are excluded or not, and anticipated regret improves product quality and is conducive to consumer rights. Atanu and Debarbata (2013) found that when piracy is prevalent, increased law enforcement efforts or equivalent piracy costs do not increase user surplus value, and our research addresses this issue [5]. We believe that this may be due to regulatory lag, so in addition to considering external regulatory factors afterwards, we also took into account internal consumer incentives beforehand. Our research findings indicate that the increase in negative utility of consumers’ expected regret towards pirated products will lead to an increase in legitimate consumer surplus. Especially under high piracy quality valuation, the rate of increase in total consumer surplus will be faster than that of legitimate consumer surplus. This also indicates that product manufacturers can build quantitative expected regret models in advance and invest them in online services. Testing and feedback of data can continuously optimize product manufacturers’ decisions and stimulate the increase of social benefits.

As shown in Fig 8, first, the increasing rate of “legal” consumer surplus in the presence of piracy is significantly faster than without piracy. This is because although anticipated regret does not increase the number of consumers who purchase genuine products, the retained consumers are those with a high willingness to pay. Therefore, their utility increases rapidly as quality improves. Second, the total consumer surplus also improves with anticipated regret, but the mechanisms are different in the two cases. Under a high piracy quality valuation, the “illegal” surplus increases due to the increase in the number of pirated users and product quality, which leads to a rapid increase in total consumer surplus than the “legal” consumer surplus. However, the negative effect of anticipated regret on individual “illegal” consumers is prominent, so the total consumer surplus rises much slower than the legal consumer surplus. Finally, given the different impacts on the entry of pirated products, whether the consumer surplus without piracy is higher than that with piracy is uncertain.

**Fig 9:**
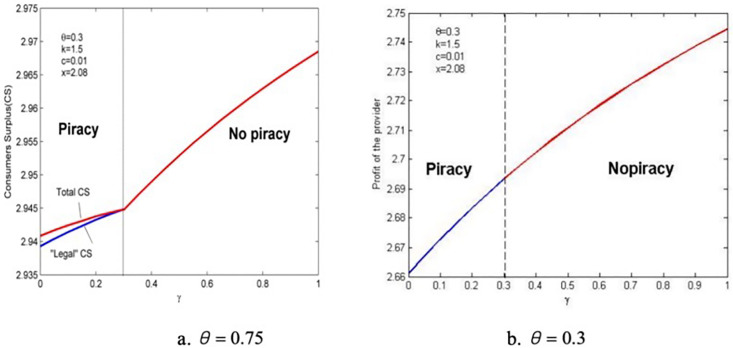
The impact of anticipated regret on the provider’s profits.

**Fig 10:**
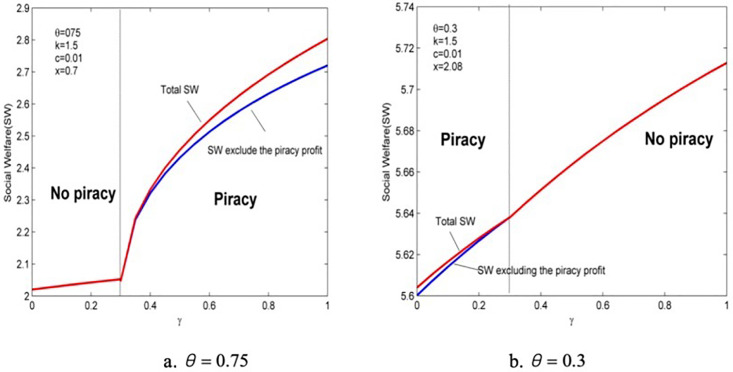
The impact of anticipated regret on social welfare.

Social welfare is the sum of consumer surplus and provider profits. Policymakers usually prefer to consider the provider’s profit and social surplus when formulating policies, so how they behave with anticipated regret is studied. Also, by excluding the profit of piracy, all profits are shown in Figs 9 and **10**.

Fig 8 shows that “moral” consumers are willing to pay a higher price for improved quality, which can compensate for the loss of demand caused by anticipated regret and R&D costs. Therefore, anticipated regret brings sure profit to the information products provider. Moreover, although the provider significantly improves product quality when piracy enters the market, it does not change the rate of growth in profits remarkably because the “crowding out” effect of piracy on genuine products will offset some of the profits.

Comparing Figs 8 and 10 it is found that the effect of anticipated regret on social welfare is entirely similar to that of consumer surplus. When consumers’ quality valuation of piracy is high, the entry of piracy can cause a significant increase in social welfare, which leads to an opposite effect of the anti-piracy goal. Thus, policymakers should not focus on an increase in consumer surplus as the primary goal of anti-piracy supervision but should devote themselves to reducing consumers’ piracy preferences. This is essential because only when consumers’ valuation of piracy is low will anticipated regret achieve the goals of driving out piracy from the market and improving product quality and social welfare.

## 7. Extensions

Current researchers (such as (Atanu and Debarbata) generally categorizes consumers into moral and non moral static categories [5,11,22], while nowadays consumers’ moral stance continuously changes with the context. Some consumers may tend to choose pirated versions in commercial software but insist on genuine versions in independent games, and their psychological decisions will change with the situation. Therefore, dynamic analysis of expected regret probability is necessary. Expected regret is a universal human decision-making psychological mechanism that combines behavioral economics and consumer psychology. Regardless of whether consumers consider themselves moral or not, they will calculate and weigh various possibilities of regret when making decisions, and the degree of regret varies among different consumers. Therefore, sensitivity analysis of expected regret is necessary.

Here, relaxing the assumption of consumer morality while assuming that buying genuine products would also lead to anticipated regret, and further studying what happens when genuine and pirated products compete at higher prices. In other words, the pirates also make decisions on *x,* which forms a three-party game model.

Now, deriving the demand function using utility analysis. The utility of consumers purchasing pirated products is presented in Formula (2). For consumers with fuzzy valuation, the probability of the anticipated regret of buying genuine products is also 0.5. In the scenario of anticipated regret, the utility of choosing (e.g., buying genuine products), $U_{c}$, is sv−p, whereas the utility of forgoing (e.g., buying pirated products), $U_{f}$, is $\theta_{H}sv-x$. Therefore, the net utility of consumers who experience anticipated regret when buying genuine products is as follows:


$$U_{g}=sv-p-\frac{\gamma }{\text{2}}\left(\left(\theta_{H}sv-x\right)-\left(sv-p\right)\right)=\frac{\left(2+\gamma \left(1-\theta_{H}\right)\right)sv-\left(2+\gamma \right)p+\gamma x}{\text{2}}$$ (14)

The demand functions of genuine and pirated products in the competitive market is as follows:


$$D_{g}=1-\frac{\left(p-x\right)}{\left(1-\theta \right)s},D_{r}=\frac{\left(2\left(1+k\right)-\left(k-1\right)\gamma \right)\theta p-\left(2\left(1+k\right)-\left(k-1\right)\gamma \theta \right)x}{\left(1-\theta \right)\left(2\left(1+k+\gamma \right)\theta -\left(1+k\right)\gamma \right)s}$$ (15)

Without losing generality, setting *c* = 2. Then, building up the model of the bilateral decision-making problem in the competitive market:


$$\begin{array}{c} \left\{ \begin{array}{c} @l@\underset{p,s}{max}\pi_{g}=\left(p-s^{\text{2}}\right)\left(1-\frac{\left(p-x\right)}{\left(1-\theta \right)s}\right)\\ \underset{x}{max\pi_{r}}=x\left(\frac{\left(2\left(1+k\right)-\left(k-1\right)\gamma \right)\theta p-\left(2\left(1+k\right)-\left(k-1\right)\gamma \theta \right)x}{\left(1-\theta \right)\left(2\left(1+k+\gamma \right)\theta -\left(1+k\right)\gamma \right)s}\right) \end{array}\right.\\ \;s.t.\;\;p>\frac{\left(2\left(1+k\right)-\left(k-1\right)\gamma \theta \right)}{\left(2\left(1+k\right)-\left(k-1\right)\gamma \right)\theta }x \end{array}$$ (16)

The equilibrium outcomes of the model are as follows:


$$\begin{array}{c} s^{*}=\frac{\text{1}}{3T},p^{*}=\frac{\text{2}}{9T^{\text{2}}},x^{*}=\frac{2-2\left(1-\theta \right)T}{9T^{\text{2}}},\\ D_{r}^{*}=\frac{2CA}{3D\left(2C-A\right)},\pi_{g}^{*}=\frac{\text{1}}{27T^{\text{2}}},\pi_{r}^{*}=\frac{4C\left(1-\left(1-\theta \right)T\right)A}{27DT^{\text{2}}\left(2C-A\right)}, \end{array}$$ (17)

where $\begin{array}{c} A=\left(2\left(1+k\right)-\left(k-1\right)\gamma \right)\theta \end{array}$, $\begin{array}{c} C=2\left(1+k\right)-\left(k-1\right)\gamma \theta \end{array}$, $\begin{array}{c} D=2\left(1+k+\gamma \right)\theta -\left(1+k\right)\gamma \end{array}$, and

$T=\frac{2\left(1+k\mathrm{\backslash rightleft}(2-\theta \right)-\left(k-1\right)\gamma \theta }{2\left(1-\theta \right)\left(2\left(1+k\right)-\left(k-1\right)\gamma \theta \right)}$. It is easy to get $\begin{array}{c} \frac{\partial T}{\partial \gamma }=\frac{\left(k^{\text{2}}-1\right)\theta }{{\left(2\left(1+k\right)-\left(k-1\right)\gamma \theta \right)}^{\text{2}}}>0 \end{array}$, then $\frac{\partial s^{*}}{\partial \gamma }<0,\frac{\partial p^{*}}{\partial \gamma }<0,\frac{\partial x^{*}}{\partial \gamma }<0,\frac{\partial {D_{r}}^{*}}{\partial \gamma }>0,\frac{\partial \pi_{n}^{*}}{\partial \gamma }<0$ is obtained.

From above, it is known that when genuine and pirated products compete in prices, the quality of information products declines with an increase in consumers’ anticipated regret sensitivity. Low-price competition of pirates is related to consumers’ anticipated regret of buying genuine products because they no longer adhere to the moral bottom line, which makes it challenging to maintain the advantageous market position of genuine products. The genuine provider must reduce the product price, which makes paying high R&D costs difficult. Therefore, unfortunately, in this situation, consumers’ anticipated regret reduces the quality of information products, reduces genuine profits, and breeds piracy, which is quite different from the scenario in which only the genuine provider decides.

**Fig 11:**
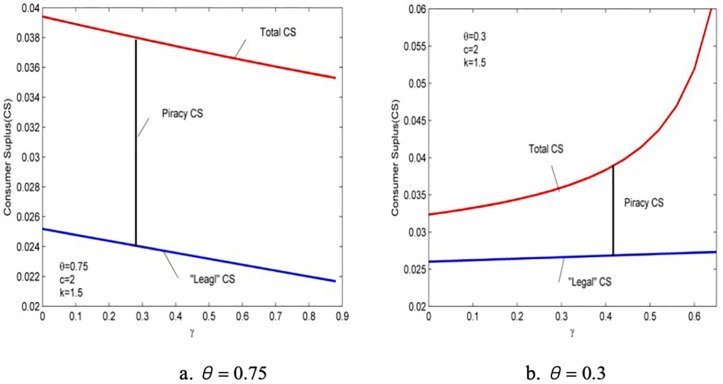
The impact of anticipated regret on consumer surplus in the extension model.

Finally, examining the consumer surplus and anticipated regret when buying genuine products under price competition, as shown in Fig 11. The figure shows that when the piracy quality valuation is low, the total consumer surplus increases significantly because the prices of genuine and pirated products decrease with anticipated regret. Thus, a reduction in the price of pirated products plays a leading role, which is proven by the rate of increase in consumers’ surplus of pirated products. However, when the piracy quality valuation is high, the two types of consumer surplus almost decline simultaneously with an increase in anticipated regret due to the significant proportion of product quality in consumer surplus, which is quite different from the basic model.

## 8. Conclusions, implications, and future works

Streaming media has driven the transformation of information products, making consumer decisions more continuous and uncertain [24–27]. Especially in the selection of information products for subscription services and cloud services, the uncertainty of quality evaluation of pirated products increases consumers’ sensitivity to expected regret, thereby changing the market entry strategy of pirated products and the quality decisions of providers [28–32]. Traditional research analyzes market access for pirated products from three aspects: consumer decision-making, product manufacturer decision-making, and market regulation [33–36]. It has been found that consumer quality preferences and enforcement costs of market regulation both have an impact on market access for pirated products [34–38]. However, in the era of streaming media, the uncertainty of information product quality and regulatory lag have exacerbated the uncertainty of consumer decision-making [39–41]. Static quality and price models are no longer able to dynamically analyze changes in consumer psychological decisions [42–44]. However, vicious price competition intensifies the passivity of genuine products, and high enforcement costs can easily lead to a decline in social benefits [45–48]. So, in order for product manufacturers to change their passive competitive situation, they must actively predict changes in consumer psychology. The quantified expected regret mechanism is an important tool for product manufacturers to predict the consumer market [49]. The dynamic use of indicators such as expected regret probability, expected regret utility, and expected regret sensitivity intuitively presents consumers’ regret psychology towards pirated information products, significantly reducing the decision-making risk of product manufacturers and promoting the overall social benefits.

This study is based on real-life case studies and constructs a quantitative expected regret model, which effectively reduces the decision-making risk of product manufacturers and mitigates the negative effects of lagging regulation. This study referred to the research of Atanu and Debarbata (2013) and found that pirated websites with zero cost downloads and pirated games purchased at low prices have put legitimate manufacturers in passive competition [5]. These types of products are mostly one-time transaction products with lower uncertainty in product quality and higher efficiency in market regulation. Therefore, consumers’ preferences for product quality and regulatory effectiveness significantly affect the decisions of product manufacturers. Nowadays, legitimate game and video software manufacturers have been continuously increasing their membership and revenue since launching subscription services. Although product manufacturers are no longer trapped in vicious price competition with pirated products, the influence of post regulatory measures is gradually decreasing. At present, genuine product manufacturers are considering taking advantage of consumers’ expected regret psychology from a forward-looking perspective to enhance the comprehensive value of genuine services and squeeze the space for piracy, and subscription system is a key strategy among them. In recent years, the revenue of digital PC games has continued to grow (with Steam being the main contributor), and the impact of piracy has relatively narrowed. The Steam platform itself is mainly based on buyout products, but has also shifted to launching subscription services, and the number of users has continued to exceed 100 million. The reason is that Steam has launched a service system that far exceeds the value of piracy, including automatic updates, cloud archiving, and frequent discounts. For example, EA Play members can enjoy better EA games with lower monthly fees, significantly increasing users’ expected regret for pirated products.

In addition to pirated games, low-cost paid downloads of pirated music have also been a cancer in the past information market. Tencent QQ Music is the largest online audio-visual music platform in China. In recent years, Tencent has also dynamically analyzed users’ expected regret utility in the decision-making process of product quality and price, and formulated differentiated subscription prices. Tencent QQ Music has launched a subscription system with different periods, including monthly, quarterly, and annual, and has also introduced higher quality super VIP members. Tencent has considered that different users have different subscription periods and varying levels of expected regret sensitivity, and has launched differentiated pricing subscriptions. The annual user subscription cycle is the longest, and the user’s sensitivity to expected regret is the highest [50]. However, the subscription price is the most favorable, so the most users choose the annual subscription. As of the second quarter of 2025, the total number of Tencent Music online music paying users is 124.4 million, of which the number of Super Membership (SVIP) users has exceeded 15 million, accounting for approximately 12.1% of the total paying users. In the first quarter of 2025, the number of paying users was 122.9 million, which increased to 124.4 million in the second quarter, with a net increase of approximately 1.5 million within six months. The proportion of subscription revenue has increased, with subscription revenue accounting for 62.4% of online music service revenue in 2023. So, these cases all indicate that product manufacturers cannot ignore the role of consumer expected regret. ‌Previous research on anti-piracy is mostly based on consumers’ deterministic valuation of piracy but ignores the effect of anticipated regret. In this study, the market demand function of pirated products based on negative utility analysis is derived. This paper also studied in detail how consumers’ anticipated regret sensitivity influences the piracy market entry strategies, price and quality decision of information providers before and after pirated products enter the market. Additionally, an extensive welfare analysis was conducted in this context.

Valuable conclusions are drew using a basic model in which only the genuine provider decides. First, if consumers’ piracy quality valuation is high, anticipated regret increases the threshold of piracy supervision and let pirated products enter the market. However, anticipated regret reduces the piracy supervision threshold, which drives piracy out of the market in the case of low piracy quality valuation. In short, with an increase in anticipated regret sensitivity, the market changes from monopoly to competition when the piracy quality valuation is high and from competition to monopoly when the piracy quality valuation is low. However, the above conclusion is valid only when the supervision (e.g., price) of piracy is within a specific range. If the supervision (e.g., price) exceeds the range, anticipated regret will not affect the market entry of piracy, and the supervision level will become the only factor that affects the market entry of piracy. These conclusions expand the research conclusion of Wang and Weng (2024) [25]. Second, it is found that the essential function of anticipated regret is to enlarge consumers’ perception of the quality of the two products and improve competitive advantage of genuine products. Thus, the provider will produce high-quality and high-price products when anticipated regret sensitivity increases, regardless of piracy entering market. However, the rate of quality improvement differs when the market changes from monopoly to competition. A competitive market can highlight the value of expected regret to product quality, especially in high piracy quality valuation.

Furthermore, under the dual influence of price and quality decisions, expected regret reduces the demand for genuine products and increases the total demand in the competitive market, resulting in a high market piracy rate. From this perspective, expected regret is not conducive to curbing piracy. However, this study found that when product manufacturers quantitatively evaluate consumers’ expected regret psychology in advance and apply it to the quality price decision-making model, the research results can be drastically different. This study referred to the research of Atanu and Debarbata (2013) and also considered the impact of market access for pirated products on social welfare [5]. Previous studies have shown that consumer quality preferences and high-intensity enforcement have an impact on market access for pirated products, but have not effectively improved social welfare. While, the welfare analysis of this research shows that whether piracy is in the market or not, the “legal” consumer surplus, total consumer surplus, and social welfare increase with anticipated regret sensitivity, which is similar to the product quality. Due to the increase in pirated users, the surplus of piracy consumers will also increase when the valuation of piracy quality is high. The provider’s profit is positively related to anticipated regret. Under the combined effects of product quality, price, and demand, a change in the rate of profits is almost the same before and after pirated products enter the market.

However, in the extension model, where genuine and pirated products compete in prices, the conclusions are quite different. It is shown that the quality and price of genuine products and profits significantly decline with the sensitivity of anticipated regret due to the low-price strategy of piracy providers. As a result, consumer surplus may also reduce in high piracy quality valuation, which is quite different from the basic model. However, when the piracy quality valuation is low, the total consumer surplus increases significantly with anticipated regret.

The conclusions have practical implications in improving the quality of information products and piracy regulation. First, product quality is the main advantage of piracy suppliers. True information product suppliers should be committed to investing in high-quality research and development, rather than just competing with piracy product suppliers based on price. Second, when consumers’ quality valuation of piracy is low, anticipated regret improves consumer surplus and is conducive to anti-piracy and product quality. So, the government should actively cooperate with providers to publicize the defects and harmfulness of pirated products in society to reduce consumers’ valuation of the quality of pirated products. Moreover, amending the law to allow unconditional returns within a certain period will attract high-risk consumers to purchase genuine products, thereby enhancing the anticipated regret sensitivity of information products. Third, the value of anticipated regret in curbing piracy can only be reflected when the supervision is within a certain range. Thus, it is suggested that the government should utilize both political supervision and consumers’ anticipated regret as anti-piracy tools. Fourth, the government needs to strengthen supervision and crackdown on piracy so that it will not be fairly priced. However, Lemma 3 indicates that when there is piracy in the market, a high piracy rate caused by a low level of piracy supervision encourages providers to invest more in quality R&D, whether consumers experience anticipated regret or not. In addition, in a competitive market, the value of anticipated regret in improving product quality and consumer surplus will be more obvious in most cases. Therefore, from a product and market perspective, it is feasible to maintain its appropriate competitive pressure on genuine products rather than eliminating piracy.

The innovation of this research is demonstrated in three aspects. firstly, the shift in research perspective, with a focus from post supervision of pirated products to pre prediction, allowing product manufacturers to shift from passive price competition to active quality optimization. Secondly, research on model optimization. Traditional quality price models in the past were unable to solve the uncertainty of information product transactions today, while quantitative expected regret models can dynamically evaluate consumer psychological changes, thereby improving the scientific nature of product manufacturers’ quality and price decisions. Thirdly, there are differences in research results. Previous studies have found that optimizing product quality, reducing market prices, and strengthening market supervision are effective measures to address piracy competition, but have not created high social benefits. However, this study found that quantitative evaluation of expected regret can avoid vicious price competition and reduce enforcement investment in market regulation, thereby effectively improving overall social welfare.

Compared with existing research, the model optimization in this study is reflected in the following three aspects:

(1) This study characterizes the purchasing utility function under consumers’ expected regret psychology. Based on the critical payment point theory, this research derives the market demand function for genuine and pirated products under expected regret. More specifically, this article applies expected regret as a behavioral parameter to the game model, and successfully optimizes the assumption of standard economic agents and enriches the practical vale of the game model.
(2) This study uses deductive reasoning to deduce how the core independent variable of “expected regret” inevitably affects the equilibrium results of a series of endogenous variables (entry, quality, and price) through rigorous mathematical logic. From this, it can be seen that this study directly constructs multiple endogenous relationships that are difficult to handle in empirical research into the model structure, avoiding the common problem of simultaneous bias in empirical research.
(3) This study conducted a joint equilibrium analysis to obtain a systematic linkage solution of market structure, price, and quality, revealing a panoramic view of the chain reaction caused by changes in a single factor. For example, the increase in consumer regret drives the improvement of product quality, which in turn promotes an increase in product prices and a decrease in product piracy rates.

The limitations for further studies are as follows. Firstly, the model did not take into account the competition among multiple genuine suppliers, and in subsequent research, the competitive relationships among multiple entities in the supply chain will be considered. Secondly, the model assumes that the two expected regret sensitivity coefficients are similar, and in future research, more in-depth factor analysis can be conducted on consumer psychology. Thirdly, in future research, empirical analysis can be combined to further demonstrate the impact of expected regret mechanisms on product manufacturers’ quality and price decisions. Finally, this study only focuses on pirated information products. Subsequent research can analyze the market access of other types of pirated products from the perspective of expected regret, and further enrich the application value of expected regret theory in solving practical problems.

## Supporting information

S1 Appendix Proof of Theorem and Lemma. (DOCX)
