# Supplementary material for: Research on the mechanism how consumer expected regret affects market access for pirated information products
Source: PLoS One. 2026 Feb 20;21(2):e0343031. doi: 10.1371/journal.pone.0343031 (PMC12923053; doi:10.1371/journal.pone.0343031)
Supplement: S1 Appendix — (DOCX) [file pone.0343031.s001.docx]

**S1 Appendix: Proof of Theorem and Lemma**

*Proof of Theorem 1:*

From the demand function of formula (5) and (6), when $p>Bx$the profit function is $\pi\left(p,s\right)=\left(p-s^{2}\right)\left(1-\frac{A\left(p-x\right)}{s}\right)$, and it is concave in *p*, then the optimal price$p^{*}\left(s\right)=\frac{s}{2A}\text{+}\frac{x+s^{2}}{2}$ is obtained from $\frac{\partial\pi}{\partial p}=0$. Substituting$p^{*}\left(s\right)$ back into $p>Bx$, the condition of high price can be obtained easily as $\frac{s}{x}\geq\left(2B-1\right)A$.

Similarly, when $p<Bx$, the profit function is $\pi\left(p,s\right)=p\left(1-\frac{p}{s}\right)-\frac{1}{2}cs^{2}$, and the first and second partial derivatives are $\frac{\partial\pi}{\partial p}=1-\frac{2p}{s}$, $\frac{\partial^{2}\pi}{\partial p^{2}}=-2/s<0$, resulting in$p^{*}\left(s\right)=\frac{s}{2}$and the condition of low price, $\frac{s}{x}<2B$, so $\frac{\left(2B-1\right)A}{2B}=A-\frac{A}{2B}=\frac{\left(1+k\right)\left(2-\theta\right)+\left(1+k-\theta\right)\gamma}{2\left(1-\theta\right)\left(1+k\right)+\left(1+k-2\theta\right)\gamma}>1$. Hence, $\left(2B-1\right)A>2B$ is always met.

Then considering the range $\left(2B-1\right)A\geq\frac{s}{x}\geq2B$. If $p\geq Bx$, $\frac{\partial\pi}{\partial p}=1-\frac{A\left(2p-x\right)}{s}<0$, we know $\pi$ is positively related with *p,* resulting in the boundary solution of $p^{\text{*}}=Bx$. If $p\leq Bx$, the profit function implies $\frac{\partial\pi}{\partial p}=1-\frac{2p}{s}>0$, and the same boundary solution of $p^{\text{*}}=Bx$. Hence, when $\left(2B-1\right)A\geq\frac{s}{x}\geq2B$ , the optimal price is simply $p^{*}=Bx$.

*Proof of Theorem 2.*

1. **No threat of piracy**. When$\frac{s}{x}<f_{1}$the profit function is $\pi\left(s\right)=\frac{s}{4}-\frac{c}{2}s^{2}$, the optimal quality level is obtained from the first-order condition, which leads to ˆ$s^{*}=\frac{1}{4c}$. Substituting it into the condition $s<s_{1}$, we know the solution is applicable if $\frac{1}{4c}<2B$, namely $x>\rho_{2}\left(\gamma\right)$.
2. **No piracy but with thereat.** When$f_{1}<\frac{s}{x}<f_{0}$the profit function of the information product provider is $\pi\left(s\right)=Bx\left(1-\frac{Bx}{s}\right)\text{ }-\frac{c}{2}s^{2}$, which is concave in *s.* Then the optimal quality level can be obtained from the first-order condition $\frac{\partial\pi\left(s\right)}{\partial s}=0$ $s^{*}=\left(\frac{B^{2}x^{2}}{c}\right)^{\frac{1}{3}}$. Substituting the substitution into $f_{0}<\frac{s}{x}<f_{1}$, it is known that the optimal quality is applicable when $\left(\frac{B^{2}x^{2}}{c}\right)^{\frac{1}{3}}\geq2Bx$ and $\left(\frac{B^{2}x^{2}}{c}\right)^{\frac{1}{3}}\leq\left(2B-1\right)Ax$are met. Solving the above two inequalities, we get $\rho_{1}\left(\gamma\right)\leq x\leq\rho_{2}\left(\gamma\right)$.
3. **Piracy entering.** As seen from lemma1, when $\frac{s}{x}>f_{0}$, there is the demand for piracy; the only possibility is that the optimal price is $\frac{s}{2A}\text{+}\frac{x+s^{2}}{2}$. The profit would be

$\pi\left(s\right)=\frac{\left(Ax+s\right)^{2}}{4As}-\frac{c}{2}s^{2}$ (A1)

and the first-order condition is $\pi^{'}\left(s\right)=0$, where

$G\left(s\right)=s^{2}-A^{2}x^{2}-4Acs^{3}=0$ (A2)

It is necessary to know the condition when the optimal *s*ˆsatisfying $s>f_{0}x$. Letting $\tilde{s}$ be the most extensive solution of $G\left(s\right)=0$. Because of $G\left(0\right)<0$ and $G\left(+\infty\right)=-\infty<0$, only $\pi^{'}\left(f_{0}x\right)>0$ will be proved so that $\tilde{s}>s_{0}$ and $\pi^{''}\left(\tilde{s}\right)<0$ are obtained. It is found that when $x<\rho_{1}\left(\gamma\right)$:

$\pi^{'}\left(s_{0}\right)>\left\{\begin{matrix} \frac{2\left(1-\theta\right)\left(1+k\right)+\left(1+k-2\theta\right)\gamma}{\left(2+\gamma\right)\left(1+k\right)} \\ *\left(1-\frac{\left(1+k+\gamma\right)^{2}\theta^{2}+\left(2+\gamma\right)\left(1+k\right)\left(2\left(1-\theta\right)\left(1+k\right)+\left(1+k-2\theta\right)\gamma\right)}{\left(\left(2-\theta\right)\left(1+k\right)+\left(1+k-\theta\right)\gamma\right)^{2}}\right) \end{matrix}\right\}>0$ (A3)

Therefore, according to the continuity of the profit function, it has at least one solution on $\left[f_{0}x,+\infty\right)$. In other words, $\tilde{s}$must exist and $\pi^{'}\left(\tilde{s}^{-}\right)>0,\pi^{'}\left(\tilde{s}^{+}\right)<0$，$\pi^{''}\left(\tilde{s}\right)<0$, implying $s^{*}=\tilde{s}$.

*Proof of Lemma 1.*

Taking the first partial derivatives of $\rho_{1}\left(\gamma\right)$ with respect to $\gamma$:

$\frac{\partial\rho_{1}\left(\gamma\right)}{\partial\gamma}=\left\{\begin{matrix} \frac{\left(2\left(1-\theta\right)\left(1+k\right)+\gamma\left(1+k-2\theta\right)\right)^{2}\left(k-1\right)\theta}{4\left(2+\gamma\right)\left(\left(2-\theta\right)\left(1+k\right)+\gamma\left(1-k-\theta\right)\right)^{3}} \\ *\left(\frac{3\theta\left(1+k+\gamma\right)}{\left(2-\theta\right)\left(1+k\right)+\gamma\left(1+k-\theta\right)}-\frac{2\left(1+k\right)\left(1-\theta\right)+\gamma\left(1+k-2\theta\right)}{\left(1+k\right)\left(2+\gamma\right)}\right) \end{matrix}\right\}$ (A4)

Whose positive and negative sign is same to $G\left(\gamma\right)=\frac{3\theta\left(1+k+\gamma\right)}{\left(2-\theta\right)\left(1+k\right)+\gamma\left(1+k-\theta\right)}-\frac{2\left(1+k\right)\left(1-\theta\right)+\gamma\left(1+k-2\theta\right)}{\left(1+k\right)\left(2+\gamma\right)}$. It can be easily seen that the former part of $G\left(\gamma\right)$ monotony decreasing and the latter monotone increasing with $\gamma$, implying $G\left(\gamma\right)$ a strictly negative correlation with$\gamma$.

Considering $0<\gamma<1$, $G\left(0\right)=\frac{3\theta}{\left(2-\theta\right)}-\left(1-\theta\right)$, so when $\theta<3-\sqrt{7}$, $G\left(\gamma\right)<0$ is always met, that is, $\rho_{1}\left(\gamma\right)$strictly negatively related with $\gamma$. Similarly, $G\left(1\right)=-\frac{2\left(2+k\right)^{2}\theta^{2}-18\left(1+k\right)\left(2+k\right)\theta+9\left(k+1\right)^{2}}{3\left(3\left(1+k\right)-\left(2+k\right)\theta\right)\left(1+k\right)}>0$, resulting $G\left(\gamma\right)>0$if $\theta>\frac{\left(9-3\sqrt{7}\right)}{2}\left(\frac{1+k}{2+k}\right)$. From the continuity and monotonicity of function $G\left(\gamma\right)$, if $3-\sqrt{7}<\theta<\frac{\left(9-3\sqrt{7}\right)}{2}\left(\frac{1+k}{2+k}\right)$, there must be a point between [0,1], $\tilde{\gamma}$. when $\gamma<\tilde{\gamma}$,$\frac{\partial\rho_{1}\left(\gamma\right)}{\partial\gamma}>0$ and when $\gamma>\tilde{\gamma}$, $\frac{\partial\rho_{1}\left(\gamma\right)}{\partial\gamma}<0$.

*Proof of lemma 3*

This proof is straightforward from the observation that $\frac{\partial s^{*}}{\partial x}>0$ from the expression of $s^{*}$ in case (1) and case (2) of theorem 2.

*Proof of Lemma 4.*

(1) **Piracy entering**. It is clear from theorem 2 that the optimal quality $s^{*}$ satisfies the first-order condition, i.e., where $s^{*}$in the interval $\left(f_{0}x,+\infty\right)$. Using the implicit function theorem, getting:

$\frac{\partial s^{*}}{\partial\gamma}=-\frac{\partial\pi^{'}\left(s\right)/\partial\gamma|s=s^{*}}{\partial\pi^{'}\left(s\right)/\partial s|s=s^{*}}$ (A5)

So,

(A6)

It is also clear from the proof of theorem 2 that $\partial\pi^{'}\left(s\right)/\partial s|s=s^{*}<0$so $\frac{\partial s^{*}}{\partial\gamma}$must be positive. Furthermore, from formula (9)， we know the optimal price,$p^{*}=\frac{s^{*}}{2A}\text{+}\frac{x}{2}$, is negatively correlated with $A$ and positively correlated with $s^{*}$, implying it is positively correlated with $\gamma$ for the relationship between $A$, $s^{*}$ between and $\gamma$ .

1. **No piracy but with thereat.** It can be seen straightforward from the first partial derivatives that:

$\frac{\partial s^{*}}{\partial\gamma}=\frac{2}{3}\left(\frac{\left(1+k\right)^{2}x^{2}\left(1+k+\gamma\right)}{4c\theta^{2}\left(2+\gamma\right)}\right)^{1/3}\frac{\left(k-1\right)}{\left(1+k+\gamma\right)^{2}}>0$ (A7)

and

$\frac{\partial p^{*}}{\partial\gamma}=\frac{\left(k-1\right)x}{2\theta\left(1+k+\gamma\right)^{2}}>0$ (A8)

When there is no piracy, the optimal quality and price are also positively correlated with the sensitivity of anticipated regret.

*Proof of Lemma 5*

From the genuine products, demand in the piracy area is given by $D\left(\gamma\right)=\frac{A\left(\gamma\right)}{2s^{*}}\text{+}\frac{1}{2}\text{ }$, which is negative with $\gamma$ since$\frac{\partial A\left(\gamma\right)}{\partial\gamma}<0$ and $\frac{\partial s^{*}}{\partial\gamma}>0$. Total demand can be expressed as $D\left(\gamma\right)+D_{r}\left(\gamma\right)=1+\frac{\left(1+k\right)L_{1}\left(\gamma\right)}{\left(2\theta\left(1+k\right)-\left(1+k-2\theta\right)\gamma\right)}$, among of which $L_{1}\left(\gamma\right)=\frac{\gamma\left(p-x\right)-2x}{s^{*}}=\gamma\left(\frac{1}{2A\left(\gamma\right)}-\frac{x}{2s^{*}}\right)-\frac{2x}{s^{*}}$, obviously $\frac{\partial L_{1}\left(\gamma\right)}{\partial\gamma}>0$ because of $\frac{\partial A\left(\gamma\right)}{\partial\gamma}<0$ and $\frac{\partial s^{*}}{\partial\gamma}>0$, implying $D\left(\gamma\right)+D_{r}\left(\gamma\right)$ increases with $\gamma$. Therefore, $\frac{\partial\left(D\left(\gamma\right)\text{/}\left(D\left(\gamma\right)+D_{r}\left(\gamma\right)\right)\right)}{d\gamma}<0$, that is$\frac{\partial\mu\left(\gamma\right)}{\partial\gamma}>0$.
